# Revisiting the NASA surface tension driven convection experiments

**DOI:** 10.1038/s41526-022-00189-5

**Published:** 2022-02-18

**Authors:** Yohan Sequeira, Abhradeep Maitra, Anupam Pandey, Sunghwan Jung

**Affiliations:** 1 Department of Biological and Environmental Engineering, Cornell University, Ithaca, NY 14853 USA; 2 Department of Mechanical and Aerospace Engineering, Cornell University, Ithaca, NY 14853 USA

**Keywords:** Fluid dynamics, Mechanical engineering

## Abstract

Marangoni effect plays an important role in many industrial applications where a surface tension gradient induces fluid flow, e.g., the cleaning process of silicon wafers and the welding process of melted metal. Surface tension gradient can also be caused by a spatially varying temperature field which, in the absence of gravity, is solely responsible for driving a large scale convective flow. NASA STDC-1 (Surface Tension Driven Convection) experiments performed on USML-1 Spacelab missions in 1992 were designed to study thermocapillary flows in microgravity. Since then these experiments have become a benchmark in thermocapillary studies in the absence of gravity. However, interpretation of results of the original STDC-1 experiments remains challenging due to the low resolution of the available data. Analysis of the velocity field in those experiments was limited to a single tracking method without systematic and comparative studies. In the present study, we utilize multiple state-of-the-art Particle Image Velocimetry and Particle Tracking Velocimetry tools to extract the flow field from NASA STDCE-1 videos and compare the experimental data to the numerical results from COMSOL Multiphysics® v5.6. Finally, we discuss how our findings of temperature-driven Marangoni flow in the microgravity setting can improve future experiments and analysis.

## Introduction

Marangoni effect^1,2^, named after physicist Carlo Marangoni^3^, is a fluid flow phenomenon driven by a surface tension gradient at a fluid-fluid interface which, in recent years, has found several industrial applications^4^ including thin film coating^5^, laser spot welding^6,7^, and surface cleaning of integrated circuit chips^8^. Manifestation of the Marangoni effect is also prevalent in our everyday world; tears of wine^9^ and fragmenting drops^10^ are some examples that captivate both scientists and children alike. Surface tension gradients in the Marangoni effect can be induced both by a temperature gradient or a concentration gradient (of a surface-active chemical) at the interface. This gradient drives a bulk convective flow which is significantly affected by the density differences across the bulk of the liquid in the case of thermocapillary flows^11^. The net convective flow is a combination of contributions from the surface tension and density gradients^12,13^. Thermocapillary flows in industrial processes usually appear in constrained geometries and with high-temperature gradients leading to flow instabilities^14,15^. The two major types of instabilities observed are—(a) Marangoni-convection instability: which is observed when the temperature difference is perpendicular to the fluid-fluid interface, and (b) thermocapillary convection instability: observed when the temperature difference is applied parallel to the interface. Therefore, a large number of studies have been focused on understanding the onset of these instabilities and the limiting conditions to avoid them^14,16,17^.

From a fundamental perspective, it is of interest to decouple the role of surface tension and gravity on the convective flow^14,18^. Naturally, performing the thermocapillary experiment in a microgravity environment was the focus of NASA in the early 90s. An interesting experimental observation, which was unexplained at that time, was the onset of an oscillatory flow, featuring temperature fluctuations at a point with an almost sinusoidal pattern and non-axisymmetric flow patterns, beyond a critical Δ*T*^19,20^. Thus, studying the properties and critical conditions for the transition from a steady to an oscillatory flow was the focus of several articles^14,21,22^. While the Marangoni number, a dimensionless quantity that compares thermal energy transport by surface tension gradient to diffusive thermal transport, was identified to play a key role in triggering the instability in experiments performed on earth and in microgravity^21,23–27^, the role of surface deformation on the onset of oscillations was unclear^21,23,28^.

In order to explore the effects of surface deformation on the oscillation onset as well as to avoid the constraints imposed by thermocapillary experiments on earth (small container to reduce gravity effects, fewer options for test fluid)^23^, Kamotani et al.^23,29^ proposed the STDC (Surface Tension Driven Convection) experiments to be conducted aboard the USML-1 spacelab in 1992^30^. The goal was to understand the effects of heating setup and free-surface shape along with the onset conditions for oscillations in the flow. Although, with the experimental conditions used (*Ma* > *Ma*_*cr*_) no oscillations were observed, the study established the fact that *Ma*_*cr*_ alone is not the criterion for oscillation onset. However, because these were some of the earliest thermocapillary flow experiments in microgravity, the analysis of the experimental videos has scope for improvement due to the development of new flow analysis tools in recent years. The initial analysis of these experimental videos utilized a particle displacement tracking method that attempted to correlate nearby particle centroids across frames. This method was able to reproduce the velocity field with reasonable accuracy but lacked in resolution due to the limited computing power available^31^. A range of more powerful particle image velocimetry (PIV) and particle tracking velocimetry (PTV) tools, such as PIVLab^32,33^, TrackMate^34^, Mosaic^35,36^, and others can be applied to the thermocapillary flow experiment videos to extract velocity fields from the experiments. In this present study, we present the comparison of seven Particle Image Velocimetry (PIV) and Particle Tracking Velocimetry (PTV) methods: TrackMate^34^ (PTV), Mosaic^35,36^ (PTV), PIVLab^32,33^ (PIV), Python PIV^37^ (PIV), Basic PIV^38^ (PIV), ImageJ Optical flow^39^ (Optical Flow), and OpenPIV-Matlab (PIV)^40^. Based on the analysis of the available NASA STDCE-1 videos^41^, we determine the most accurate tracking method for extracting velocity fields from the STDCE-1 experiment videos. We show an improvement over the analysis method of Wernet et al. in 1991^31^ by increasing the spatial and temporal resolution of the extracted velocity field with reduced processing time. Finally, we compare our results with simulation results obtained from COMSOL Multiphysics® v5.6 and make recommendations on improvements to future experiments.

## Results

### Measured velocities from PIV and PTV tools

**Fig. 1:**
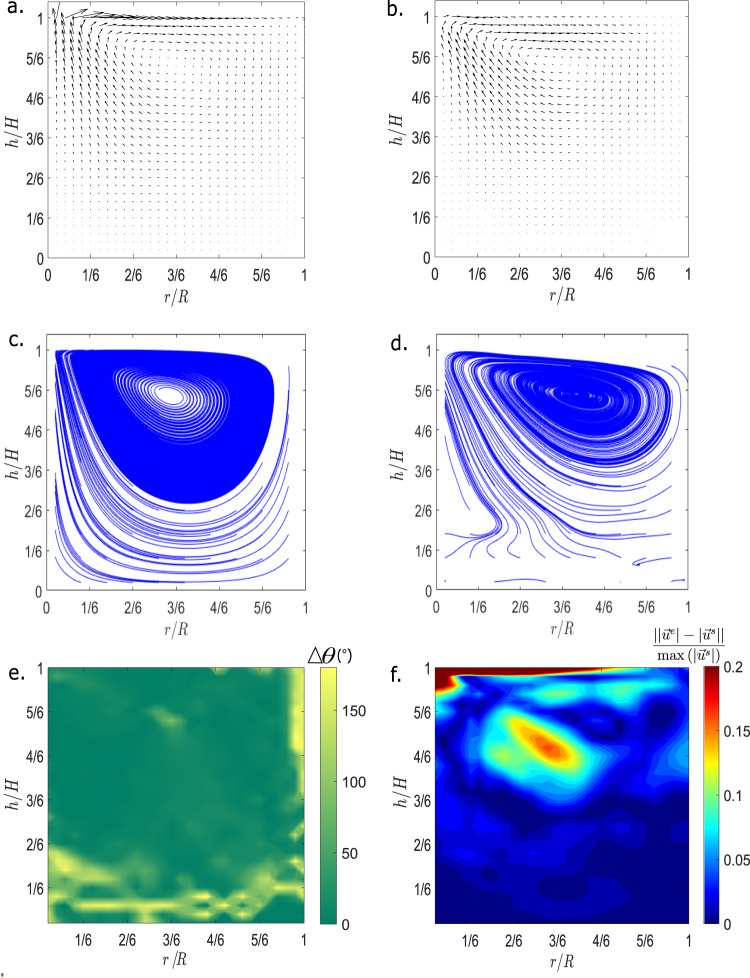
Comparison of PTV and simulation velocity fields of Run 1 CT1 (Pr = 96, Ma = 46,000). **a** Generated velocity field from simulation in COMSOL MultiPhysics®. **b** Extracted velocity field from PTV method TrackMate. **c** Plotted streamlines for simulation velocity field shown in **a**. **d** Plotted streamlines for PTV velocity field shown in **b**. **e** Angle difference in degrees (Δ*θ*) between the PTV and Simulation velocity vectors. **f** Normalized magnitude difference $\left(\frac{\left|\left|{\overrightarrow{u}}^{e}\right|-\left|{\overrightarrow{u}}^{s}\right|\right|}{\max \left(\left|{\overrightarrow{u}}^{s}\right|\right)}\right)$ between the PTV and Simulation velocity fields, where ${\overrightarrow{u}}^{e}$ represents experimental velocity vector and ${\overrightarrow{u}}^{s}$ represents simulated velocity vector.

Seven different PIV and PTV analysis tools generated velocity vector fields. Each of these calculations is averaged over 200 frames of video, each frame downsampled from the original 30 frames per second to a six frame-per-second sequence, in order to increase the apparent particle velocities between the analyzed frames. The resulting video sequences comprise around 1.3 min of experiment time. The frames used for this analysis are sampled from the steady-state period of the experiment, which was reached after 10 min of the start of the experiment^23,30^. In order to compare the various methods, several measures are calculated. We first calculate the streamlines, as a way to qualitatively compare the PIV and PTV methods and the numerical simulations, as shown in Fig. 1c, d. The extracted streamlines are somewhat offset in their vertical direction, which may be an artifact of the skew correction. Furthermore, small differences in the velocity fields (see Fig. 1a, b) can induce large differences in streamline shapes. Thus, the streamline data provide only a qualitative comparison between the extracted experimental velocity field and the simulation velocity field.

A quantitative comparison between generated vector fields is the two-dimensional divergence of flow. It is calculated as:

1 $$| \nabla \cdot \overrightarrow{u}| =\left|\frac{d{u}_{x}}{dx}+\frac{d{u}_{y}}{dy}\right|$$

In two-dimensional flow, we expect that the divergence becomes zero at every point in the field. A physically accurate PIV/PTV method outputs a vector field with a zero value of divergence at each point in the velocity vector field. Assessing which method outputs a flow field with low absolute divergence is a way to compare methods across the same representative experiment. Data for a representative experiment (Run 1 CF2) are shown in Table 1, which shows the mean and standard deviation values of divergence for the seven different methods.

**Table 1:** Compared values of the mean (plus or minus standard deviation) of divergence, and the mean (plus or minus standard deviation) of normalized difference in angle between PTV and simulation velocity fields for the seven different PIV methods across a single representative video. (Run 1 CF2).

Method name	Mean of divergence (1/s)	Mean of normalized angle difference
TrackMate	0.026 ± 0.01	0.21 ± 0.05
PIVLab	0.20 ± 0.14	0.37 ± 0.06
Python PIV	0.5 ± 0.04	0.28 ± 0.07
OpenPIV	0.19 ± 0.21	0.33 ± 0.07
Mosaic	0.006 ± 0.005	0.32 ± 0.1
OpticFlow	0.04 ± 0.01	0.7 ± 0.06
Basic PIV	0.27 ± 0.49	0.23 ± 0.07

**Fig. 2:**
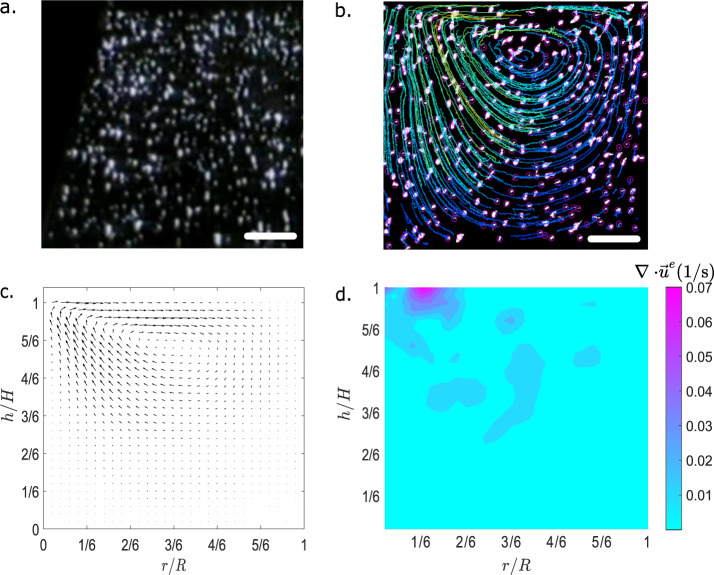
Analysis of tracked video of Run 1 CT1 (Pr = 96, Ma = 46,000). **a** View of the cropped experimental video before skew correction and thresholding (Left-Side). **b** Skew Corrected video after tracking, blue and green lines represent the final overlaid particle tracks while purple circles represent bounding shapes of detected particles. Note that the tracked data are flipped across the vertical axis in relation to the orientation of the original video. **c** Extracted velocity field from TrackMate after track-processing and smoothing. **d** Computed divergence values for the extracted velocity field $(\nabla \cdot {\overrightarrow{u}}^{e})$, where ${\overrightarrow{u}}^{e}$ represents experimental velocity vector. Scale bars (**a**, **b**), 1 cm.

A representative plot of the divergence is shown for the method TrackMate in Fig. 2d. As displayed, the overall divergence is quite low (0.045 s^−1^), and larger values are visible near the upper surface where both the magnitude and angle of the flow field change rapidly over a small region. In such regions, PIV and PTV methods may overestimate velocity values.

Since TrackMate and Mosaic give low divergence fields as well as a good qualitative streamline comparison, we choose these two methods for further comparison with the numerical calculations. The direct comparison between experiment and numerical simulation helps us identify the regions of vanishing velocity field in the PIV/PTV measurements that lead to a low value of divergence but a high magnitude of the error. Figure 1 shows an example of a direct comparison between numerical simulation and PTV results in one of the representative cases. Figure 1a, b shows the extracted velocity fields for the numerical simulation velocity field data and the extracted PTV velocity, respectively. Figure 1c, d shows the streamline data for the numerical simulation velocity field data and the extracted PTV velocity, respectively. Figure 1e shows the angle difference between the simulation and PTV velocity field data, and Fig. 1f shows the normalized magnitude difference between the same data. The normalized magnitude difference is calculated as shown in the equation below:

2 $$| | {\overrightarrow{u}}^{e}(x,y)| -| {\overrightarrow{u}}^{s}(x,y)| | /\max (| {\overrightarrow{u}}^{s}| ),$$

where the absolute magnitude of the velocity at each point in the simulation is subtracted from the absolute magnitude of the velocity extracted from the PIV/PTV data, and then normalized by the maximum of the simulated velocity across the entire flow field. The highest normalized magnitude difference is found near the top left corner of the plot. In the top left corner, the velocity magnitude of the flow field is the highest and the free surface is directly above. In this region, tracking methods are unable to capture the high velocity and rapid change of direction of particles. A similar effect can be seen near the edges and center of the vortices, and near the corners of the flow field. Inaccuracy in the tracking methods in these regions leads to a velocity mismatch between the PIV velocity data and the simulated velocity field, and ultimately cause a large error.

**Fig. 3:**
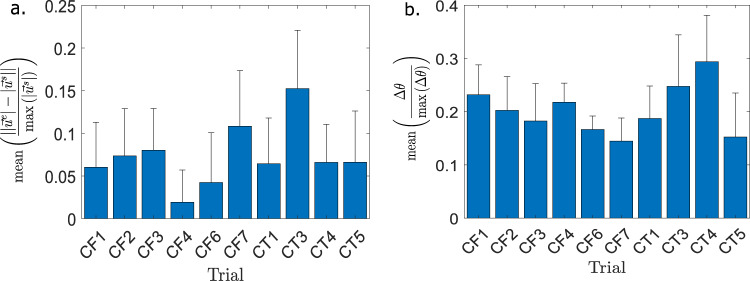
Comparison of velocity difference across different methods. **a** A comparison of the mean of the absolute normalized difference in velocity magnitude, between the PTV and simulation results, across the analyzed videos for the analysis method TrackMate. Magnitude difference values are calculated after the first ten minutes of the experiment. **b** A comparison of the mean of the absolute normalized difference in angle across the analyzed videos for the analysis method TrackMate. Angle difference values are calculated after the first ten minutes of the experiment.

Figure 3 shows the mean value of the normalized magnitude and angle difference between the extracted velocity field and the simulation velocity field over different experiments. The mean magnitude difference between simulation and velocity vector fields is generally around 5% for most experiments, despite a significant standard deviation. In most cases, the bulk of the low-velocity fluid matches in magnitude between experiment and simulation. The significant difference in magnitude appears near the free surface. In contrast, the angle difference is around 20% between simulation and extracted data in the Run 1 experiments. This is likely due to good angle matching between the velocity field near the surface of the flow field and near the vortices, but a poor match in the bulk of the flow. As the flow of fluid decreases near the bottom of the experimental container, it is likely that the tracking algorithms have more difficulty determining the direction of the flow, leading to a greater angle difference (see the colormap of Fig. 1e). In this lower region reflections and bubbles likely also have a detrimental effect on angle matching. Across all the experiments, TrackMate is out-performed by other algorithms such as PIVLab and Mosaic in velocity field magnitude accuracy in the high-velocity regions, but performs better in tracking fluid velocity angle and magnitude in the bulk of the fluid flow. In all PIV/PTV methods tested, the maximal difference in angle occurs primarily near the bottom edges of the flow field. This is likely caused by the fluid being obscured by reflections in the bottom surface of the tank, which in turn introduce error to the PIV and PTV methods.

**Fig. 4:**
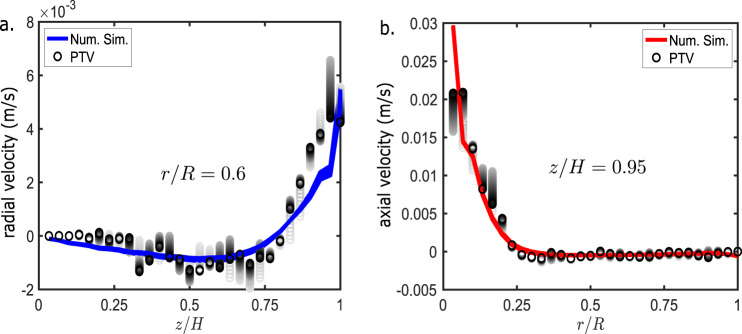
Comparison of radial and axial velocities from PTV and simulation results for Run 1 CF1. **a** Radial velocity along *z* at *r*/*R* = 0.6. Circles are radial velocity from PTV analysis (lighter colors are used for earlier times in the experiment). **b** Vertical velocity along *r* at *z*/*H* = 0.95. Circles are axial velocity from PTV analysis (lighter colors are used for earlier times in the experiment).

Following the comparison plots used in Kamotani et al.^23,42^, we have done a similar comparison of near-surface velocity and bulk velocity at specific planes between the PTV data and the COMSOL simulation. This comparison was conducted only for the flat-surface cases, as variations in the curved surface made the automated computation of surface velocity field difficult. Figure 4 shows the comparison between the experimental (PTV) and the simulation velocity at the distance of *h*/*H* = 0.95 for the axial velocity and at *r*/*R* = 0.6 for radial velocity. The colored lines show the numerical velocity profiles between 10 and 60 min of experimental time, whereas the circles represent experimental data. The gradient color coding is used to show changes in data points over time. The axial velocity along *z*/*H* = 0.95 and the radial velocity along *r*/*R* = 0.6 show a good match between PTV and simulation results. Some deviations of experimental results from simulation may be attributed to the difficulty of the PTV methods in tracking particles within the moving vortex, present near the center of the flow field, where the direction of particles changes rapidly in a small area.

In our analysis of the STDCE-1 experiments, the bulk of the error comes from cropping, skew correction and edge effects. PIV errors are caused due to reflections created by the light on the bottom surface of the container in the experimental setup. The skewed viewing angle of the video also introduces a skew error in the final velocity fields, despite the applied correction step. Finally, the presence of bubbles also causes a significant error, especially in cases where the videos were taken over a short time span. Qualitatively, our extracted experimental velocity fields and simulation results compare reasonably well with the original PIV and simulation results in the previous papers^23,30,42^.

### Improvements from previous work

Due to a loss of the original data, it is impossible to perform a quantitative comparison with previously published results. We can, however, comment on how our tracking methods improve on the original methods which Kamotani et al.^23^ used. Primarily, our method improves upon the spatial and time resolution of velocity field extraction. Our analysis (on average) is able to produce three times as many velocity vectors per frame analyzed than the original method, which produced 1992 vectors per frame^31^. Furthermore, the previous method was limited in it’s sub-pixel tracking, leading to velocity errors of up to 18% in regions where the fluid velocity was low. Finally, we compare various methods for the extraction of velocity fields, while the original analysis was limited to a single analysis method due to lack of availability and computational restraints.

## Discussion

Based on our analysis, we propose the following improvements to any future STDC or similar thermocapillary flow experiments. Firstly, an increase in seed particle density would allow for finer resolution PTV analysis, especially in regions such as the swirling vortices and the center of the flow field, where the fluid velocity changes rapidly in both magnitude and direction. Secondly, the low viewing angle at which the video is taken requires significant skew correction of the experimental videos in order to create a rectangular vector field for comparison with simulated results. This skew correction introduces significant error in the upper region of the velocity field. Finally, revisions of the experimental apparatus could be conducted to mitigate the effects of large bubbles in the fluid and reflections of the laser on the container, which adversely affect the PTV results. The effect of bubbles and reflections are most prevalent in shorter tests.

Based on the results of each of the PIV and PTV methods, we can also comment on the type of analysis methods that are best suited for analyzing STDCE experiments. The successful methods were able to accurately track a wide range of particle speeds. In the STDCE experiments, the velocity of particles at the interface is much larger compared to the velocity of particles near the bottom of the container. Tracking methods failed to capture this range of velocities when they were unable to link corresponding particles across frames, so robust interframe tracking is necessary. Also important is how robust the method is to artifacts. The provided STDCE videos have significant artifacts, such as bubbles in the fluid and reflections of the laser. Such artifacts introduce significant numbers of erroneous vectors, especially in single-step methods. PTV methods such as TrackMate are resilient to optical artifacts (specifically those which appear for only a few frames) because extraction of a velocity vector at a point is found by computing the velocity of tracked particles through a region, unlike PIV methods which track optical flow through a specific region of the image. Based on these findings, the best methods for the analysis of future STDCE videos would be methods with a high interframe linking distance, low sensitivity to experimental artifacts and fast computational time.

An open question in the surface tension driven convection experiments is how interfacial curvature affects the resulting fluid flow. No definite answer has been found from the previous analysis of the experimental videos, possibly due to the limited number of curved surface conditions which could be created on USML-1 SpaceLab^23,25^. Future experiments utilizing an improved experimental setup and up-to-date PIV and PTV methods (as well as simulation techniques) could shed light on accurately analyzing Marangoni flows to understand many industrial drying, cleaning, and welding processes on earth.

## Methods

### Description of STDC in-space experiments

**Fig. 5:**
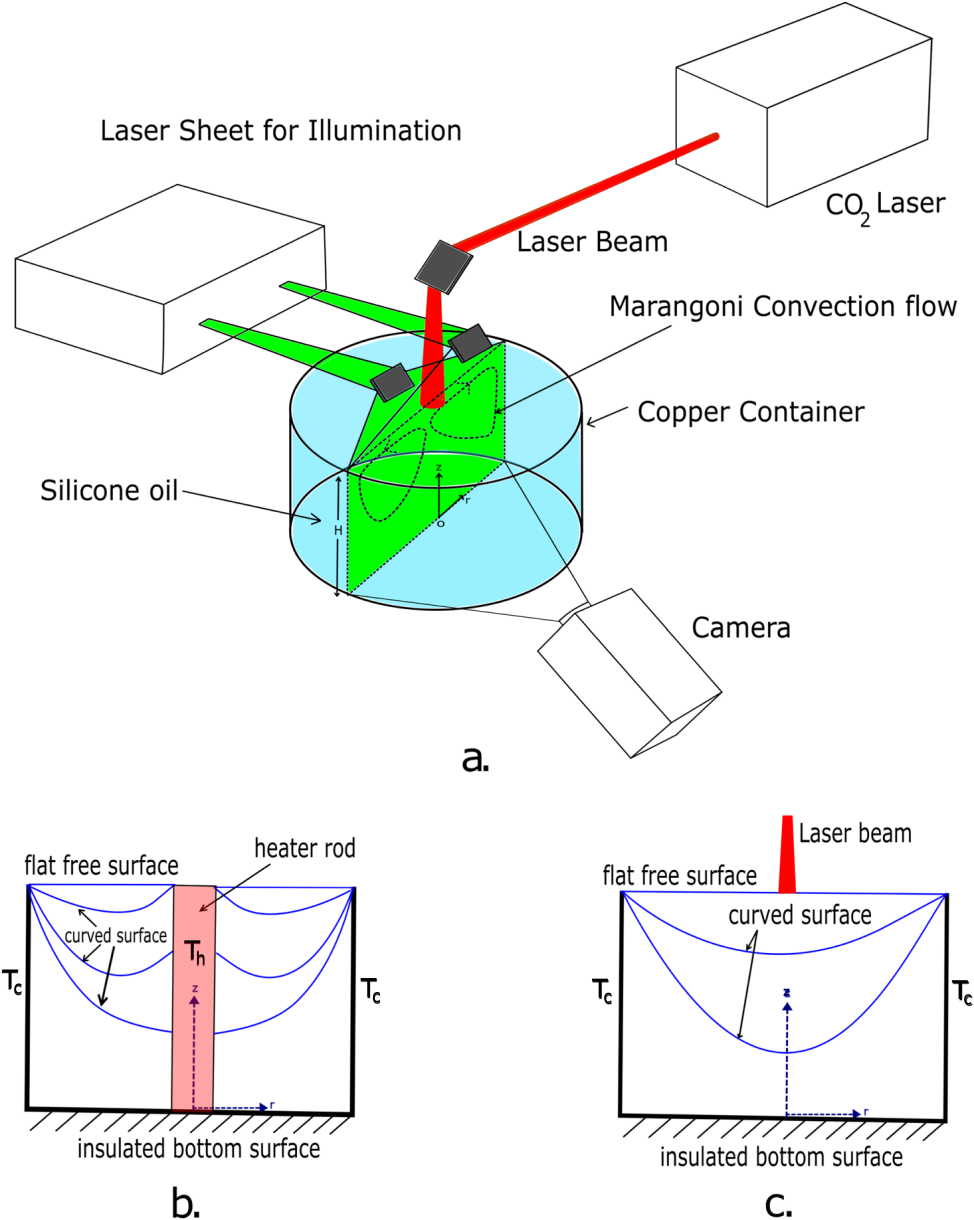
Schematics of experimental setup. **a** An angled view of the simplified STDC experimental setup used on the 1992 USML-1 spacelab mission. A CO_2_ laser (red) is used to heat the silicone oil shown in the copper container (blue). A second, low power laser (green) is used to create a laser sheet which illuminates the Marangoni-convection flow. A camera records the experiment from below, looking through the clear bottom of the container. **b** A side cross-section of the experiment using a heater rod (CT). The heater rod (red) is inserted in the center of the cylindrical tank to create a constant temperature at the center and induce flow. The bottom of the tank is insulated. Curved blue lines show different curved surface configurations. **c** A side cross-section of the experiment using a heating laser (CF). The laser beam shines into the center of the tank to generate a constant heat flux at the center and induce the Marangoni flow. The bottom of the tank is insulated. Curved blue lines show different curved surface configurations.

A schematic of the NASA STDC experimental setup used in the 1992 USML-1 Spacelab mission is presented in Fig. 5a. Experiments were performed within cylindrical copper containers with a Teflon base. The containers had a depth of 5 cm and a diameter of 10 cm, and were filled with silicone oil. The outer walls of the containers were kept at a constant temperature using a heat exchanger, while a resistive heating rod, placed at the center of the container (1.1 cm diameter shaft) heated the silicone oil and induced a constant temperature field as shown in Fig. 5b. To impart a constant flux condition, the heating rod was replaced by a CO_2_ laser that pointed at the center of the container (see Fig. 5c). Curvature of the oil-air interface was also varied by changing the volume of silicone oil in the container and by pinning the contact line at the outer wall to a sharp lip at the top of the container. Both temperature and velocity fields were measured throughout the experiments. The oil temperature was recorded using temperature rakes placed throughout the tank and on the central heating rod. The accuracy of the temperature measurements was found to be less than 1% of the overall temperature difference in the liquid. The velocity field was measured using a particle image velocimetry (PIV) technique in which 50 μm alumina particles were illuminated by a 1 mm-thick laser sheet to visualize the flow field. A CCD camera captured the image through the bottom (flat) surface of the tank, to eliminate the lensing (keystone) effect. Further experimental considerations are described by Kamotani and Ostrach^21^, whereas flight hardware configurations are reported by Pline et al^29^.

There are four main conditions under which the experiments were performed: the constant temperature (CT) case with either flat or curved interfaces and the constant flux (CF) case with either flat or curved interfaces. The system is fully described by the following ten-dimensional variables: tank radius (*R*), tank height (*H*), the radius of the heating zone or the heating rod (*R*_*h*_), the velocity of the liquid (*v*), the density of the liquid (*ρ*), surface tension (*γ*), the dynamic viscosity of the liquid (*μ*), thermal diffusivity of the liquid (*α*), temperature difference along with the interface (Δ*T*) and the temperature coefficient of surface tension (*σ*_*T*_). In the constant temperature tests, Δ*T* is defined as the difference in temperature between the heater and the sidewall. In the constant flux tests, Δ*T* is defined as the difference between the maximum fluid temperature and the sidewall temperature. For curved interfaces, the additional parameter of the contact angle at the tank wall becomes relevant. Under experimental conditions it was determined that in-flight experiments would take about ten minutes to reach a near steady-state flow and temperature profile^43^.

### Pre-processing experimental movies

We analyze an exemplary set of videos from the list of all experiments given in Kamotani et al.^30^—run 1 CF1, run 1 CF2, run 1 CT1, and run 4 CT2. The nomenclature is identical to the nomenclature used in the report by Kamotani et al.^30^. Run 1 CF1 and Run 1 CT1 were both hour-long tests, and were noted in the previous video analysis as being tests in which the flow was allowed to reach steady flow conditions^21,23^. Run 1 CT1 and run 1 CF2 incorporated the heating element and Co2 laser respectively. Run 4 CT2 was used as it was a 30 min curved surface test. We start the analysis of STDC videos by correcting for the skew, which resulted from misalignment between the viewing angle of the camera and the plane of the laser sheet. To correct this skew, we utilize MATLAB® to apply a correction matrix to each frame. The same correction matrix is applied to all videos to maintain consistency. When applied to the image frames, the correction matrix transforms the images by smoothly upscaling (stretching) the upper portion of the frame, and downscaling (shrinking) the lower portion. The frames are transformed such that the aspect ratio of the frame matches with the ratio of the borders of the PIV sheet in the experiment. We also condition the movies for the PIV/PTV algorithms by applying simple sharpening and image dilation filters to remove blurring and other undesirable artifacts. Figure 2 shows half of a frame of video before skew correction and sharpening is applied, whereas Fig. 2b shows the same half-frame after skew correction and sharpening with particles tracks overlaid. After skew correction and sharpening, videos are spliced and only the portions of the experiments with steady flow conditions are used for the PIV analysis. For experiments that are shorter than the ten minutes required to reach a steady flow, the entire video is analyzed. The final step of pre-processing involves subsampling of the videos. The videos are subsampled at five frames per second, reducing the number of total frames from the original video rate of thirty frames per second. This reduction is performed to increase the magnitude of displacement of particles between frames in relation to the PIV window size being used.

### PTV and PIV methods

Different PTV and PIV methods, as listed in Table 2, are tested to determine which gave the best results. The PIV methods chosen differ somewhat in implementation, but all use the same underlying principles to determine velocity fields from the video frame data. PIV methods generally split a video of fluid motion into multiple small sections known as windows, in which at least six to ten particles are visible^44,45^. Cross-correlation techniques such as histogram matching or optical flow algorithms are used to match the relative movements of windows (and the particles within them) between frames^33,38,44^. By sequentially decreasing the size of the correlation windows throughout iterations of the algorithm, velocity fields can be generated with a finer resolution^38,44^. In methods such as PivLab^32,33^ and OpenPIV^40^ additional options such as vector validation are available in post-processing. In the vector validation step, erroneous vectors can be smoothed and filtered using interpolation methods. PTV methods are a two-step process, consisting of an object detection stage and an object tracking (or frame linking) stage^34,36^. In the object detection stage, computer vision or statistical methods are used to identify particles or objects in each video frame^46,47^. In the object tracking or frame linking stage, the detected objects are linked across frames to create a sequence of locations of the object in the video frame over time^34,36^. The instantaneous velocity of each tracked object is calculated from the displacement of a particle between frames and dividing this displacement by the time step between frames. The resulting velocity value can be assigned to the midpoint of the particle locations between the two tracked points^34,36,47^.

**Table 2:** PIV methods used to analyze the select exemplary videos.

Method Name	Type
TrackMate	PTV
PIVLab	PIV
Python PIV	PIV
OpenPIV-Matlab	PIV
Mosaic	PTV
OpticFlow	Optical flow
Basic PIV	PIV

The PIV and PTV methods tested are of three main categories: pure optical flow methods, integrated optical flow and vector validation methods (PIV) and particle tracking methods (PTV). The final seven methods tested are selected for several traits. One trait was the variability in implementation. Methods were chosen such that they utilized a wide range of strategies to track particles or estimate velocity fields. For example, the PTV algorithms chosen (Mosaic and TrackMate) both employ a two-step tracking method to identify particles and track them across frames. Mosaic and TrackMate both use optimization methods to determine the most likely trajectories of particles, but differ in their initial particle detection algorithms. Similarly, all PIV methods use optical flow to measure the displacements between windows, but differ in the precise implementation of the algorithm as well as the vector validation and interpolation implementation. Furthermore, methods such as PIVlab and TrackMate offer a multi-step process by which tracks or vectors are filtered and re-analyzed, whereas the optical flow method provides a single-step process.

### Numerical analysis

For comparison to the extracted PIV and PTV velocity vectors, numerical simulations for all the NASA STDC experimental cases have been performed on COMSOL Multiphysics® v5.6 using Multiphysics couplings (a. Non-isothermal flow, b. Marangoni effect) between the Laminar Flow and Heat Transfer in Fluids modules. The boundary conditions and parameter values used for the numerical simulations have been borrowed from the NASA STDC experiments described above in the sub-section titled description of STDC in-space experiments^30^. The model used here is based on the numerical model developed by Kamotani et al.^42^ for analysis of the NASA STDC experiments. Based on the setup geometry (as shown in Fig. 5) an axisymmetric cylindrical coordinate system (*r*, *θ*, *z*) is used for the numerical simulations here. As described above in the sub-section titled description of STDC in-space experiments, the experiments performed were surface tension driven flows due to temperature gradients in a microgravity environment. In order to model the phenomena, the following governing equations have been included in the model

3 $$\frac{Ma}{Pr}\left(\frac{\partial \overrightarrow{u^{\prime} }}{\partial t^{\prime} }+\overrightarrow{u^{\prime} }\cdot \nabla \overrightarrow{u^{\prime} }\right)=-\nabla p^{\prime} +{\nabla }^{2}\overrightarrow{u^{\prime} }$$

4 $$Ma\left(\frac{\partial T^{\prime} }{\partial t^{\prime} }+\overrightarrow{u^{\prime} }\cdot \nabla T^{\prime} \right)=-\nabla \overrightarrow{q^{\prime} }+\overrightarrow{Q^{\prime} },$$

which are the non-dimensionalized momentum and thermal energy conservation equations respectively, where $Ma=\frac{{\sigma }_{T}{{\Delta }}TR}{\alpha \mu }$ is the Marangoni number, $Pr=\frac{\nu }{\alpha }$ is the Prandtl number, $\overrightarrow{u^{\prime} }=\frac{\overrightarrow{u}}{{u}_{c}}$, $t^{\prime} =\frac{t}{{t}_{c}}$, $p^{\prime} =\frac{p}{{p}_{c}}$, $T^{\prime} =\frac{T-{T}_{c}}{{{\Delta }}T}$, $\overrightarrow{q^{\prime} }=\frac{\overrightarrow{q}R}{k{{\Delta }}T}$, $\overrightarrow{Q^{\prime} }=\frac{\overrightarrow{Q}{R}^{2}}{k{{\Delta }}T}$, ${u}_{c}=\frac{{\sigma }_{T}{{\Delta }}T}{\mu }$, ${t}_{c}=\frac{R}{{u}_{c}}$, ${p}_{c}=\frac{\mu {u}_{c}}{R}$, *ρ* is density of the fluid, *T*_*c*_ is the temperature of the curved wall (lower temperature), *C*_*p*_ is specific heat capacity of fluid, $\overrightarrow{u}$ is velocity vector, *p* is pressure, *μ* is dynamic viscosity of the fluid, *k* is the thermal conductivity of the fluid, *T* is temperature, $\overrightarrow{q}$ is heat flux in the fluid, and $\overrightarrow{Q}$ is energy added from a heat source.

Based on the experimental setup (as shown in), the hydrodynamic boundary conditions used are -

1. No-slip (at side and bottom walls of container)
  5 $$\overrightarrow{u^{\prime} }-(\overrightarrow{u^{\prime} }\cdot \overrightarrow{n})\overrightarrow{n}=0$$
  where $\overrightarrow{n}$ is the normal vector to the wall.
2. No penetration (at all boundaries)
  6 $$\overrightarrow{u^{\prime} }\cdot \overrightarrow{n}=0$$
3. Slip at top free surface
  7 $$\overrightarrow{u^{\prime} }-(\overrightarrow{u^{\prime} }\cdot \overrightarrow{n})\overrightarrow{n}\,\ne\, 0$$
4. Shear stress balance at free surface (Marangoni boundary condition)
  8 $$(\bar{\bar{\tau }}^{\prime} \cdot \overrightarrow{n})\cdot \overrightarrow{{t}_{i}}=({\nabla }_{s}\gamma ^{\prime} )\cdot \overrightarrow{{t}_{i}}$$
  where ${\nabla }_{s}=\nabla -\overrightarrow{n}(\nabla \cdot \overrightarrow{n})$, $\bar{\bar{\tau }}^{\prime} =\frac{\bar{\bar{\tau }}^{\prime} R}{\mu {u}_{c}}$, $\bar{\bar{\tau }}$ is stress tensor, $\overrightarrow{{t}_{i}}$ are the orthogonal directions on the interface plane, $\gamma ^{\prime} =\frac{\gamma }{{\sigma }_{T}{{\Delta }}T}$ and *γ* is the surface tension of the liquid.

Based on the fluid properties used in Kamotani et al.^42^ and the NASA STDC report^30^, the temperature coefficient of surface tension is *σ*_*T*_ = −5.5 × 10^−5^ *N*/*m* ^∘^C and the dynamic viscosity is modelled using the function $\mu /{\mu }_{0}=1-1.71\times 1{0}^{-2}(T-{T}_{r})+1.06\times 1{0}^{-4}{(T-{T}_{r})}^{2}$ where *μ*_0_ = 9.4 × 10^−3^ Pa ⋅ s. The density variation with temperature is not considered because the experiments were conducted in a microgravity environment. Based on the in-space experiments, the thermal boundary conditions used are broadly of two different types -

1. Constant temperature (CT tests)—The constant temperature setup has a heating rod of diameter 1.11 cm inserted into the cylindrical container at the center. The heating rod is uniformly maintained at a constant higher (than walls and surrounding air) temperature thus providing a constant temperature boundary condition at $r^{\prime} =0.11=Hr$, where *Hr* = *R*_*h*_/*R* is defined as the relative heater ratio. The parameters which are varied in the CT experiments are the temperature difference between the heater rod and curved wall (Δ*T*), the total time of the experiment and the surface curvature. Although the experimental data shows that the heater takes some time (1–2 min) to reach a steady-state temperature, here we have used a constant temperature for the heater rod, which is equal to the steady-state temperature.
2. Constant flux (CF tests)—The constant flux setup has a CO_2_ Laser beam pointed on the free surface. The centre of the Laser beam is aligned with the centre of the free surface. Due to the geometric symmetry of the fluid domain about the centre line, a no flux condition is used as the boundary condition at $r^{\prime} =0$. The laser enters the fluid domain and gets attenuated as it passes through the fluid. Thus, the energy in the beam is transferred to the fluid layers and gradually decreases the beam intensity with depth. Therefore, as the laser adds heat to the fluid bulk and not just the surface, it is incorporated as a heat source term in the thermal energy conservation equation. The Laser-beam modelling adopted here is based on the model in Kamotani et al.^42^ and the details of the modelling can be found in section Laser-beam modelling.

The other thermal boundary conditions common to both setups are—

1. Constant temperature at curved wall—
  9 $$T^{\prime} =0$$
  Similar to the heating rod, instead of using the temperature variation overtime data for the walls of the container, average temperatures have been used for constant temperature boundary conditions due to unavailability of the NASA experimental temperature variation data and mostly small variations in temperature within experimental timescale. (nearly 1–2 °C).
2. No Flux condition at the bottom wall
  10 $$\overrightarrow{n}\cdot \nabla T^{\prime} =0$$
3. At the free surface, a complete energy balance equates the conduction of heat from the bulk of the liquid to the sum of the convective heat flux from the liquid to the fluid above and a radiation flux from the surface to the surrounding^48^. As the NASA STDC experiments were performed in microgravity conditions, the contribution of the convection term due to natural convection is negligible compared to the radiation term in the equation^42^. So, finally, for the flat free-surface cases, we have the following heat flux boundary condition at the free surface^42^—
  11 $$\overrightarrow{n}\cdot \nabla T^{\prime} =-4Ra(T^{\prime} -{T^{\prime} }_{{{{\rm{amb}}}}})$$
  where $Ra=\frac{\epsilon \sigma {{T}_{{{{\rm{amb}}}}}}^{3}H}{k}$ is the radiation parameter, a non-dimensional parameter introduced following the numerical analysis in refs. ^30,42^, *ϵ*(=0.9)^42^ is the emissivity of fluid surface, *σ* is the Stefan-Boltzmann constant and $\overrightarrow{n}$ is the unit normal at the point. The equation in the curved surface cases includes an extra term due to view-factors between surface elements on the curved surface (discussed below in sub-section titled Curved surface heat flux boundary condition).

### Laser-beam modelling

Following Kamotani et al.^42^, the Laser is modelled as a Gaussian beam with a general intensity distribution given by—

12 $$I(r)={I}_{0}{e}^{(-2{r}^{2}/{r}_{b}^{2})},$$

where ${I}_{0}=\frac{2{P}_{0}}{\pi {r}_{b}^{2}}$ is the peak intensity, *P*_0_ is the beam power and *r*_*b*_ is the beam radius. The beam diameter is assumed to remain constant inside the fluid domain because most of the intensity gets attenuated within a small distance (attenuation length is 0.06 mm here^29,30^ and the change in the beam diameter within that small distance is negligible. Considering the effect of beam attenuation, the intensity distribution inside the fluid domain is given by—

13 $$I(r,z)={I}_{0}{e}^{(-2{r}^{2}/{r}_{b}^{2})}{e}^{-a(H-z)},$$

where the second exponential multiplier captures the decay in intensity due to absorption by the fluid, *H* is the height of the cylindrical container and *a* is the attenuation coefficient which is the reciprocal of the attenuation length. Attenuation length is defined as the length within which the intensity falls to 1/*e* times of the original intensity^29^. In order to calculate the heat source term, which is the rate of heat energy added per unit volume of the fluid, we consider a fluid element at a position (*r*, *z*) and write a heat balance equation as follows—

14 $$Q{^{\prime\prime\prime}} dV=[I(z+dz,r)-I(z,r)]dA,$$

where the term on LHS is the amount of heat energy added to the element per unit volume, the first term on RHS is the incident heat energy from the laser beam and the second term is the amount of heat energy transmitted to the next fluid element. The difference in the incident and transmitted heat energy is absorbed by the fluid element itself and is captured using the heat source term given by—

15a $$Q{^{\prime\prime\prime}} =\mathop{\lim }\limits_{dz\to 0}\frac{I(z+dz,r)-I(z,r)}{dz}$$

15b $$\Rightarrow Q{^{\prime\prime\prime}} =\frac{2a{P}_{0}}{\pi {r}_{b}^{2}}{e}^{(-2{r}^{2}/{r}_{b}^{2})}{e}^{-a(H-z)}.$$

Unlike the constant temperature case where the curved surface experiments did not require any changes to the equations other than just defining the free surface in the geometry based on the free-surface shapes given in the NASA report^30^, the constant flux case needs modification in the heat source term because of two reasons—

1. The beam diameter during the NASA experiments was measured at the location of the free surface for the flat-surface case. The location of the free surface for curved surface cases is not at the same height. Therefore, the beam diameter for the curved surface cases is recalculated based on the data provided on the change in beam diameter with axial distance^30^.
2. As the surface is curved, the beam enters the fluid at different heights depending on the radial position therefore the exponential decay term for attenuation needs to be modified accordingly.

The height at which the beam enters the fluid is given by

16 $$L(r)=H\left(1+\tan \alpha -\frac{\cos ({\sin }^{-1}(r\cos \alpha /H))}{\cos \alpha }\right),$$

where *α* is the angle made by the tangent to the curved surface at the outer edge and the vertical wall (values of which are given in the NASA report, P. 37). Incorporating *L*(*r*) and the new beam diameter (*r*_*b*,*new*_) into the heat source equation, the modified heat source term for the curved surface cases is—

17 $$Q{^{\prime\prime\prime}} (r,z)=\frac{2a{P}_{0}}{\pi {r}_{b,new}^{2}}{e}^{(-2{r}^{2}/{r}_{b,new}^{2})}{e}^{-a(L(r)-z)}.$$

### Curved surface heat flux boundary condition

In the curved free-surface cases, the radiation term in the heat flux boundary condition (Eq. (11)) will have an extra irradiation contribution due to the view-factors between surface elements. The heat flux boundary condition can be derived from the general form of the equation for diffuse and gray surfaces^48^ as follows—

18a $$\overrightarrow{n}\cdot \overrightarrow{q}=\epsilon \sigma {T}^{4}-\alpha \left({G}_{amb}+\mathop{\sum}\limits_{S}{G}_{ot}\right)$$

18b $$\Rightarrow \overrightarrow{n}\cdot \overrightarrow{q}=\epsilon \sigma {T}^{4}-\alpha \sigma {\epsilon }_{amb}{F}_{amb}{T}_{amb}^{4}-\alpha \mathop{\sum}\limits_{S}\epsilon \sigma {F}_{j}{T}_{j}^{4}$$

where *α* is the absorptivity of the liquid free surface, *G*_*amb*_ is the part of the irradiation from the ambient, *G*_*ot*_ is the part of the irradiation from other surface elements, *ϵ*_*amb*_ is the emissivity of the ambient, *F*_*amb*_ is the view-factor representing the fraction of radiation emitted by the ambient which is received by the current surface element, *T*_*amb*_ is the temperature of the ambient, *F*_*j*_ is the view-factor representing the fraction of the total radiation emitted from the jth surface element intercepted by the current surface element, *T*_*j*_ is the temperature of the jth surface element. The summation in the last term is over all the surface elements. To incorporate the view-factors, the surface-to-surface radiation module was used in COMSOL Multiphysics ® v5.6. The Hemicube method (COMSOL Multiphysics ® v5.6 Heat Transfer Module) was used for the simulation to include the view-factors between surface elements on the curved free surface.

### Reporting summary

Further information on research design is available in the Nature Research Reporting Summary linked to this article.

## Supplementary information

Reporting Summary

## Data Availability

All data and plots generated during this study are in the Open Science Framework (OSF) repository, 10.17605/OSF.IO/XGS7R.

## Code availability

The custom codes used in the study are available from the corresponding author on reasonable request.
